# The Divergent Effects of Nicotinamide Riboside and High‐Intensity Exercise Training on Skeletal Muscle Epigenetic Aging

**DOI:** 10.1111/acel.70638

**Published:** 2026-07-21

**Authors:** Aino Heikkinen, Liina Uusitalo‐Kylmälä, Ida Blom, Jørn Wulff Helge, Linn Gillberg, Robert Seaborne, Steen Larsen, Macsue Jacques, Robin Grolaux, Sari Aaltonen, Jaakko Kaprio, Birgitta W. van der Kolk, Sini Heinonen, Nir Eynon, Kirsi H. Pietiläinen, Riikka Kivelä, Eija Pirinen, Miina Ollikainen

**Affiliations:** ^1^ Minerva Foundation Institute for Medical Research Helsinki Finland; ^2^ Institute for Molecular Medicine Finland (FIMM), HiLIFE University of Helsinki Helsinki Finland; ^3^ Faculty of Sport and Health Sciences University of Jyväskylä Jyväskylä Finland; ^4^ Department of Biomedical Sciences University of Copenhagen Copenhagen Denmark; ^5^ Laboratory of Sports and Nutrition Research Riga Stradiņš University Riga Latvia; ^6^ School of Basic and Medical Biosciences King's College London London UK; ^7^ Clinical Research Centre Medical University of Bialystok Bialystok Poland; ^8^ Australian Regenerative Medicine Institute, Faculty of Medicine, Nursing and Health Sciences Monash University Clayton Victoria Australia; ^9^ Obesity Research Unit, Research Program for Clinical and Molecular Metabolism, Faculty of Medicine University of Helsinki Helsinki Finland; ^10^ Department of Internal Medicine Helsinki University Hospital Helsinki Finland; ^11^ HealthyWeightHub, Endocrinology, Abdominal Center Helsinki University Central Hospital and University of Helsinki Helsinki Finland; ^12^ Wihuri Research Institute Helsinki Finland; ^13^ Stem Cells and Metabolism Research Program, Faculty of Medicine University of Helsinki Helsinki Finland; ^14^ Research Unit of Biomedicine and Internal Medicine, Faculty of Medicine University of Oulu Oulu Finland; ^15^ Research Program for Clinical and Molecular Metabolism, Faculty of Medicine University of Helsinki Helsinki Finland; ^16^ Medical Research Center Oulu Oulu University Hospital, University of Oulu Oulu Finland; ^17^ Biocenter Oulu University of Oulu Oulu Finland

**Keywords:** epigenetic aging, high‐intensity interval training, mitochondria, nicotinamide riboside, skeletal muscle, twins

## Abstract

Aging is accompanied by a decline in physiological function and increased vulnerability to disease, with mitochondrial dysfunction and epigenetic alterations recognized as key hallmarks. Nicotinamide riboside (NR), a vitamin B3 precursor to NAD^+^, and high‐intensity interval training (HIIT) have both been proposed to ameliorate aging‐related mitochondrial decline, but their effects on skeletal muscle epigenetic aging are not fully elucidated. Here, we assessed the impact of 5‐month NR supplementation and 4–6 weeks HIIT on epigenetic age acceleration (EAA, via seven epigenetic clocks) in human skeletal muscle across three independent studies. NR supplementation was associated with reduced muscle EAA, particularly when measured with the PCHannum, MEAT, and DunedinPACE clocks, while HIIT produced opposite effects in some clocks, notably increasing pace of aging by DunedinPACE. Correlation analyses revealed that changes in skeletal muscle mitochondrial content correlated with changes in MEAT‐derived EAA after NR and 6 weeks of HIIT. Together, these findings indicate that skeletal muscle epigenetic aging can be modulated by NR and HIIT interventions but in opposing directions, highlighting a potential link between mitochondrial abundance and epigenetic clocks. Further studies are warranted to clarify how NR and exercise regulate epigenetic aging. These results offer new insights into development of strategies for promoting epigenetic outcomes and healthy aging.

## Graphical abstract

This study investigated the effects of nicotinamide riboside supplementation and high‐intensity interval training on skeletal muscle epigenetic aging. Our findings reveal that both interventions may modify epigenetic aging, with the muscle‐specific MEAT and DunedinPACE clocks showing the most responsiveness and links to mitochondrial abundance.

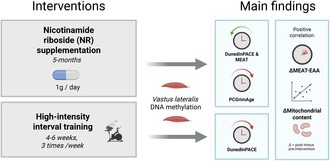

## 1 Introduction

Human aging is characterized by the progressive accumulation of cellular damage, leading to functional decline across tissues, increased disease burden, and ultimately death. Although aging affects all organs, skeletal muscle is particularly critical due to its role in mobility, metabolic health, and quality of life. Age‐related deterioration of skeletal muscle contributes to sarcopenia and frailty, markedly increasing the risk of falls, disability, and mortality in older adults (Xu et al. 2021). Many hallmarks of aging are tightly intertwined with cellular metabolism (López‐Otín et al. 2023), in which the mitochondrion is a central organelle known for converting chemical energy into ATP. Unsurprisingly, mitochondrial dysfunction is recognized as one of the hallmarks of aging. Aged skeletal muscle shows lower mitochondrial number and DNA (mtDNA) abundance, citrate synthase (CS) activity, and diminished expression of mitochondrial‐related genes (Short et al. 2005). These alterations are further linked to age‐related changes such as chronic inflammation, oxidative stress, and impaired nutrient signaling.

NAD^+^ and its related metabolites NADH, NADP and NADPH maintain cellular redox balance, and NAD^+^ itself also serves as a consumed substrate for several enzyme families such as poly(ADP)ribose polymerases (PARPs), sirtuins and cluster of differentiation 38 (CD38). These enzymes are involved in processes like epigenetic regulation, mitochondrial metabolism, inflammation and oxidative stress, thereby linking NAD^+^ metabolism to multiple hallmarks of aging (López‐Otín et al. 2023). Evidence suggests that NAD^+^ homeostasis may be altered with age in a tissue‐specific manner, potentially affecting also skeletal muscle (Covarrubias et al. 2021). This has prompted interest in whether strategies to restore NAD^+^ levels could extend healthspan and delay onset of age‐associated diseases. Two primary strategies have emerged to boost NAD^+^ levels: pharmacological supplementation with, for instance, NAD^+^ precursors and lifestyle interventions such as exercise training (Covarrubias et al. 2021).

Among NAD^+^ precursor vitamin B3s, nicotinamide riboside (NR) has been shown to be safe in humans and effective in increasing NAD metabolite levels both in blood (Lapatto et al. 2023) and in skeletal muscle (Elhassan et al. 2019). In rodent models, NR supplementation improves skeletal muscle mitochondrial content and respiration, mitigates age‐related muscle dysfunction and extends lifespan (Cantó et al. 2012; Zhang et al. 2016), although contrasting results are also reported (Shi et al. 2017). Findings are also mixed in humans, particularly regarding whole‐body metabolic outcomes. Some studies have reported modest improvements in inflammatory markers (Brakedal et al. 2022) while others have found no beneficial effects on adiposity or insulin sensitivity (Dollerup et al. 2018; Lapatto et al. 2023). Furthermore, the effect of NR in skeletal muscle function remains understudied. Notably, we have previously shown that 5‐month NR supplementation increases skeletal muscle mitochondrial content, decreases global DNA methylation and modifies methylation of CpG sites in mitochondria‐related genes in monozygotic twins (Lapatto et al. 2023). Overall, while promising effects of NR have been observed, the evidence remains inconsistent, likely due to differences in intervention duration, participant characteristics, and specific outcome measures.

Exercise training is another promising booster of NAD^+^ metabolism. Both aerobic and resistance exercise training have been shown to increase muscle NAD^+^ levels and the expression of muscle nicotinamide phosphoribosyltransferase (*NAMPT*) (de Guia et al. 2019), the primary rate‐limiting enzyme in NAD^+^ biosynthesis in muscle. Exercise also induces broad metabolic benefits, including improved mitochondrial function and insulin sensitivity (Cartee et al. 2016). High‐intensity interval training (HIIT) is a time‐efficient exercise modality that improves cardiorespiratory fitness and metabolic health faster than traditional endurance training (Ko et al. 2025). It has also been shown to increase NAMPT expression and NAD^+^ levels in blood (Walzik et al. 2025) and induce DNA methylation changes associated with a younger epigenetic profile (Ostaíza et al. 2025; Voisin et al. 2024).

Despite these advances, it remains unclear whether NR supplementation or HIIT can slow epigenetic aging as measured by epigenetic clocks. Epigenetic age represents a composite measure of cumulative aging‐associated changes in DNA methylation patterns over time. These measures are biomarkers that associate with many aging‐related symptoms, such as functional decline, disease, and mortality. Numerous epigenetic clock algortihms have been developed, each capturing distinct biological signatures: first‐generation clocks (e.g., Horvath, Hannum, and the muscle‐specific MEAT) were primarily trained to predict chronological age, whereas second‐generation clocks (e.g., GrimAge, PhenoAge) and pace‐of‐aging measures (DunedinPACE) incorporate physiological data and aging‐associated biomarkers to better reflect underlying biological processes (Teschendorff and Horvath 2025). This diversity of the clocks highlights the need to understand how different clocks respond to specific physiological stimuli, particularly in tissues like skeletal muscle where data remain limited, to screen the potential of these biomarkers for evaluating anti‐aging strategies in the future. To address this, we assessed six widely used blood‐based epigenetic clocks (as applied, e.g., in Li et al. 2026), and MEAT (Voisin et al. 2020, 2021), the only muscle‐specific clock, to evaluate their responsiveness in parallel to NR and HIIT because both modify skeletal muscle biology through mitochondrial function and NAD‐related pathways, providing a relevant framework to assess how such interventions may influence muscle epigenetic aging.

In this study, our aim was to conduct a preliminary, exploratory evaluation of how widely used epigenetic clocks respond to NR and HIIT interventions in skeletal muscle. We also examined whether intervention‐related changes in these clocks might reflect underlying biological processes, including alterations in mitochondrial content or inflammation. Specifically, we assessed whether 5 months of NR supplementation in the Finnish Twin Cohort (Kaprio 2013) and short‐term HIIT interventions in the EpiH (Søgaard et al. 2018) and Gene SMART (Yan et al. 2017) studies influence epigenetic aging estimates in skeletal muscle. Although these studies were not originally harmonized for direct comparison, our goal was to provide initial insight into whether these two distinct interventions modulate epigenetic age in similar or divergent ways. We additionally evaluated the effects of NR on blood epigenetic aging, as blood is the primary tissue in epigenetic aging research and the main target for most epigenetic clocks. Together, these exploratory analyses reveal that NR and HIIT may influence epigenetic aging in opposing directions, with responses varying by individual and by clock. These findings provide a foundation for understanding how different interventions affect epigenetic biomarkers and highlight the inherent variability across clocks, tissues, and individuals.

## 2 Materials & Methods

### 2.1 Study Cohorts

#### 2.1.1 5‐Month Human NR Trial

The human NR trial was originally performed to investigate the effect of 5‐month NR supplementation (with the final dose of 1000 mg/day) on muscle and adipose tissue mitochondrial biogenesis (clinicaltrials.gov entry NCT03951285) using monozygotic twin pairs, targeting twin pairs discordant for body mass index (BMI). These pairs originate from three larger longitudinal Finnish Twin Cohorts (FTC; FinnTwin12, FinnTwin16, and Older Finnish Twin Cohort) (Kaprio 2013). The full study design and exclusion and inclusion criteria are described in detail in the original study paper (Lapatto et al. 2023).

In the current study, we utilized data on adult twin individuals (30–65 years) who were administered NR supplementation with available DNA methylation samples from both timepoints (before and after NR) from either of the two tissues: blood (*n* = 36 individuals) and muscle (*n* = 30 individuals). We included twin individuals without any data from their co‐twin to increase the number of samples in each analysis that was performed at the individual level. For the comparison between the influence of NR on epigenetic ages between leaner and heavier co‐twins, we included only complete BMI‐discordant twin pairs (*n* = 14 and 15 pairs in muscle and blood, respectively) defined as having a minimum difference of 2.5 BMI units between co‐twins.

Venous blood and skeletal muscle samples (*Vastus lateralis*) were collected from each participant at baseline and after 5‐months of NR supplementation (Lapatto et al. 2023). The muscle biopsies were collected using a Bergström needle under local anesthesia, snap‐frozen and stored in liquid nitrogen, and then transferred to −80°C. The blood samples were stored at −80°C. High‐molecular weight DNA was extracted from muscle and blood samples using the QIAmp DNA Mini kit (Qiagen) and bisulfite‐converted by an EZ DNA Methylation kit (ZYMO Research) for the quantification of DNA methylation levels.

The measurements of whole‐blood NAD^+^ and related metabolites (NAD^+^, NADP, NMN, NAR, NAAD, ADPR and Me4Py) were performed as described by Lapatto et al. (2023).

#### 2.1.2 EpiH Study

The EpiH samples are derived from an exercise training intervention study in which 20 younger (21–42 years) and 20 older (55–74 years) individuals with overweight or obesity (BMI range 27–44 kg/m^2^) were recruited (Søgaard et al. 2018). The inclusion criteria as well as the detailed study protocol are described elsewhere (Søgaard et al. 2018). Briefly, the individuals completed a 6‐week supervised HIIT intervention three times a week on a bicycle ergometer. Each HIIT session consisted of 2 min warm up followed by five 1‐min intervals at 124% ± 12% of their maximal load (W) interrupted by 90 s of cycling at 25 W or just resting on the bike. After 2 weeks of training, the load was increased by 10%. An incremental test on a bicycle ergometer was performed before and after the intervention to determine maximal oxygen uptake during exercise (VO_2max_) which reflects cardiorespiratory fitness. Two VO_2max_ tests were performed on two separate test days before the HIIT intervention to avoid potential learning effects.

Muscle biopsies from the *vastus lateralis* were collected prior to (maximum 1 week) and after the intervention (48–72 h after the final HIIT session) using a Bergström needle and manual suction in an overnight fasted condition (Søgaard et al. 2018). Muscle tissue was immediately snap‐frozen in liquid nitrogen and thereafter stored at −80°C. The frozen tissue was treated with 2 mg/mL (0.2%) collagenase ( *Clostridium histolyticum* , Type I, Sigma‐Aldrich) and myofibers were extracted manually to enrich myofibers and to remove non‐myofiber material. Genomic DNA was extracted from the washed and frozen down pellet of sorted myofibers using the DNeasy Blood and Tissue Kit (Qiagen, Maryland, USA).

#### 2.1.3 Gene SMART Study

The Gene SMART (Skeletal Muscle Adaptive Response to Training) study is a multi‐center exercise training study aiming to identify OMIC biomarkers of the response to 4 weeks of HIIT (Jacques et al. 2020). To date, 100 male and 30 female young (18–48 years old), healthy, moderately‐fit participants have undergone four‐week supervised HIIT intervention on an electronically braked cycle ergometer. The detailed Gene SMART methods and study design can be found elsewhere (Yan et al. 2017; Jacques et al. 2023). Briefly, the 4‐week HIIT program included three supervised sessions per week. Each session was preceded by a 5‐min warm‐up at 50 W, followed by six to twelve two‐minute intervals at intensities ranging progressively from 40% to 70% above the individual's lactate threshold, interspersed with 1‐min recovery periods. VO_2max_ was assessed before and after four interventions using a protocol consisting of 2‐min exercise bouts performed to exhaustion.

Muscle samples were collected from the *vastus lateralis* before and after (48 h after the last session) HIIT intervention using a Bergström needle and manual suction. The samples were frozen in liquid nitrogen and stored at −80°. Approximately 15 mg of homogenized bulk muscle tissue was used for DNA extraction with the Qiagen AllPrep DNA/RNA kit.

### 2.2 Myotube Cell Culture

Primary human myoblasts were plated into 6‐well plates (~120,000 cells/well) and when the cells reached 80%–90% confluency (typically within 2–4 days), a differentiation medium was introduced to promote maturation into myotubes. Cells were cultured in the differentiation medium for 6 days. Following the differentiation, the cells were exposed to a medium containing 1 mM of NR (Novalix Pharma, Strasbourg, France) (*n* = 6 technical replicates) or a control medium without NR (*n* = 6 technical replicates). A concentration of 1 mM NR has been shown to produce the maximal increase in intracellular NAD^+^ levels in C2C12 myotubes (Cantó et al. 2012) and is typically the highest concentration used in in vitro experiments. Due to the rapid degradation of NR, the medium was replaced daily. After 72 h of exposure, cells were harvested for analysis. The contents of the cultivating media are described in Data S1.

For DNA extraction, we used the AllPrep DNA/RNA/miRNA Universal Kit (Qiagen) according to the manufacturer's instructions, with the modification that DNA was eluted using 2 × 50 μL of RNase‐free water. To measure NAD^+^ levels, cells were first washed with PBS. Subsequently, three wells were pooled together into an Eppendorf tube, and the cell pellet was collected following centrifugation at 1400 rpm for 10 min. The quantification of NAD^+^ levels was performed using the NADMED assay (Euro et al. 2025).

### 2.3 Epigenetic Ages

#### 2.3.1 DNA Methylation Data Preprocessing

DNA methylation data (.idat files) from skeletal muscle across all studies (human NR trial, myotube cell culture, the EpiH cohort, and the Gene SMART cohort; GEO accession: GSE171140) and from blood in the NR trial only were normalized and preprocessed using the R (*meffil* package) following a pipeline described in detail elsewhere (Heikkinen et al. 2025). Methylation data from the human NR trial were generated using the Illumina Infinium MethylationEPIC v1 array, while all other DNA methylation data were generated using the EPIC v2 array.

#### 2.3.2 Epigenetic Clocks

Epigenetic age estimates were calculated from the preprocessed DNA methylation beta values using PC‐based versions of the original Hannum, Horvath, PhenoAge, and GrimAge clocks (Higgins‐Chen et al. 2022). In addition, we computed the pace of aging measure DunedinPACE (Belsky et al. 2022), GrimAge2 (Lu et al. 2022), as well as the muscle‐specific MEAT clock for DNA methylation data derived from muscle tissue (Voisin et al. 2020). Both PCGrimAge and the more recent GrimAge2 were included since the PC‐based version may reduce susceptibility to technical noise, whereas GrimAge2, still lacking a PC‐based version, incorporates two additional plasma‐based biomarkers in the prediction. In addition, the updated MEAT clock, the MEAT2 (Voisin et al. 2021), was used for all other studies except for the Gene SMART, which was used in the development of MEAT2, and therefore the original MEAT1 (Voisin et al. 2020) was applied instead. Epigenetic age acceleration (EAA) was defined as the residuals from regressing epigenetic age estimates on chronological age and was calculated separately for each study. Outliers, defined as values exceeding ±3 standard deviations from the study mean EAA, were excluded from downstream analyses. In the NR trial, one outlier was identified for muscle (GrimAge2) and three for blood (one in DunedinPACE, two in GrimAge2). In EpiH, there were no outliers, whereas in Gene SMART, two outliers were detected with the GrimAge2 clock.

### 2.4 Mitochondrial Content

Relative mitochondrial DNA quantity (mtDNAq) was assessed in the NR trial muscle biopsies using quantitative PCR (qPCR) from phenol‐chloroform extracted DNA. The mtDNA amount was determined by quantifying three mitochondrial genes—*D‐Loop*, cytochrome b (*CYTB*), and 16S rRNA relative to three nuclear reference genes: amyloid beta Precursor Protein (*APP*), Beta‐2‐Microglobulin (*B2M*), and Hemoglobin Subunit Beta (*HBB*). The data were processed using qbase+ software (Biogazelle) by standard curve method. The average calibrated normalized relative quantities of the mitochondrial target genes were used to calculate mtDNAq for each sample.

In cultured myotubes, mtDNAq was similarly measured using qPCR, targeting the mitochondrial genes NADH Dehydrogenase Subunit 5 (*ND5*) and *CYTB*, normalized to the nuclear genes *APP* and *B2M*. The mean of the *ND5* and *CYTB* CNRQ measures was used to represent mtDNAq for each sample.

In HIIT studies, measurements of the citrate synthase (CS) activity were used as markers for the mitochondrial content in the skeletal muscle. In EpiH, the CS activity was calculated using spectrophotometry from 2 mg freeze‐dried and dissected muscle tissue as described elsewhere (Frandsen et al. 2022). In Gene SMART, small pieces of freeze‐dried skeletal muscle tissue were lysed in an ice‐cold buffer (KH_2_PO_4_ & K_2_HPO_4_) and total CS activity was measured in triplicates using spectrophotometric assays (Jacques et al. 2021).

### 2.5 Statistical Analysis

All the analyses were performed in R statistical software (version 4.5.1).

#### 2.5.1 Performance of Epigenetic Clocks in Predicting Chronological Age

We used Pearson correlation to evaluate how well epigenetic clocks that provide age estimates in years, excluding DunedinPACE, predict chronological age. To quantify the average deviation between predicted and actual age, we calculated the mean absolute error (MAE).

#### 2.5.2 Before Versus After Intervention

Differences in EAA before and after NR supplementation or HIIT were assessed using linear mixed‐effects models (*lmerTest* R package) adjusted for chronological age, sex, and BMI. In the human NR trial, models were additionally adjusted for self‐reported smoking status. To account for repeated measures, models included random effects: unique participant identifiers for the HIIT studies and participant ID nested within family ID in the NR trial to account for both repeated measures and relatedness of twins in a pair.

In the myotube experiment, differences in EAA between control and NR‐treated cells were analyzed using linear models. Changes in NAD^+^ levels and mtDNAq were assessed using the Wilcoxon signed‐rank test.

To investigate whether leaner and heavier co‐twins in BMI‐discordant twin pairs differed in EAA changes following NR, we calculated pre vs. post EAA changes and used linear mixed effects models to assess the effect of weight status (i.e., lean or heavy), adjusting for sex, smoking and family ID.

#### 2.5.3 Intra‐Class Correlation

The intra‐class correlation coefficients (ICCs) were estimated using linear mixed‐effects models with a random intercept for family ID, with either the baseline EAA or change in EAA upon NR as an outcome. For the analysis of change in EAA, mean baseline EAA within a twin‐pair was included as a covariate to estimate within‐family resemblance in change of EAA upon NR independent of baseline values. The 95% confidence intervals for the ICC estimates were obtained using parametric bootstrapping with the *bootMer* function in R, based on 1000 simulated datasets drawn from the fitted models.

#### 2.5.4 Correlation Analyses

The correlations between delta variabes (i.e., post‐intervention minus pre‐intervention values within an individual) of different EAA clocks and mtDNAq in the NR trial, and of different EAA clocks, CS activity and VO_2max_ in EpiH and Gene SMART studies were estimated using Pearson correlation. Additionally, in the NR trial, the correlation between EAA and mtDNAq within twin pairs was assessed using Pearson correlation on the difference in changes between co‐twins (i.e., delta–delta variables, calculated by subtracting the change in twin 1 from the change in twin 2).

## 3 Results

**FIGURE 1:**
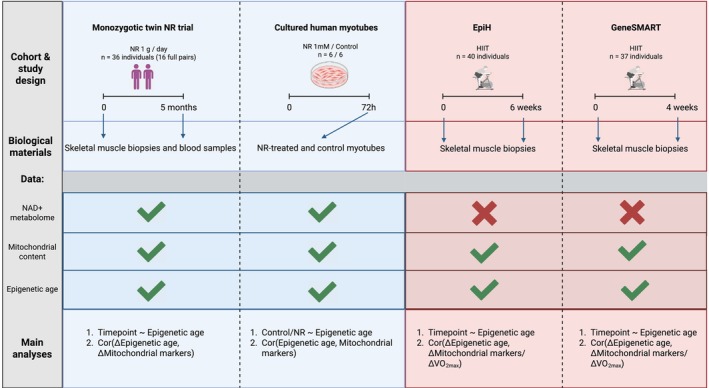
Schematic representation of the study design, datasets, and statistical analysis for each study. Created with Biorender.com.

Figure 1 illustrates the study design, and Table 1 summarizes the participant characteristics of the three human studies. Mean ages ranged from 33 years in Gene SMART to 40 in NR study and 48 in EpiH, while female participants comprised 44%–48%. The mean baseline BMI was 24.4 kg/m^2^in the Gene SMART, whereas NR and EpiH studies included predominantly individuals with overweight or obesity (mean baseline BMIs 30.3 and 32.0 kg/m^2^, respectively).

**TABLE 1:** Characteristics of participants included in this study.

	FTC NR trial		EpiH		Gene SMART	
Reference to the study design	Lapatto et al. 2023		Søgaard et al. 2018		Yan et al. 2017	
Intervention	Nicotinamide riboside (NR) supplementation 1000 mg/day 5 months		HIIT intervention on a bicycle ergometer three times/week Six weeks		HIIT intervention on a bicycle ergometer three times/week Four weeks	
Age at baseline, years	40.0 (8.9)		47.7 (16.5)		33.5 (8.3)	
*Mean (SD) range*	29.6–65.3		21.0–74.0		19.0–47.0	
Sex, *% female*	44.4		47.5		48.7	
Timepoint	Before	After	Before	After	Before	After
Number of samples^a^ *Blood, muscle*	36, 32	35, 30	0, 40	0, 39	0, 37	0, 37
BMI, kg/m^2^	30.3 (5.8)	31.2 (6.1)	32.0 (3.8)	31.9 (3.9)	24.4 (3.2)	24.5 (3.4)
*Mean (SD) range*	20.1–44.7	20.4–45.6	27.4–42.6	26.6–42.7	18.4–32.0	18.8–32.2
VO_2max_, ml/min/kg *Mean (SD)*	NA	NA	26.6 (5.3)	28.5 (6.0)	46.5 (8.3)	46.6 (7.4)
CS activity, μmol/min/g	NA	NA	127.6 (36.0)	161.1 (39.0)	193.3 (77.1)	216.6 (89.5)

Abbreviations: BMI = body mass index, CS = citrate synthase, FTC = Finnish Twin Cohort, HIIT = high‐intensity interval training, NA = not available, VO_2max_ = maximal oxygen consumption.

^a^ After DNA methylation data quality control.

### 3.1 Performance of the Epigenetic Clocks

Because the epigenetic clocks, except the muscle‐specific MEAT clock, were originally developed for blood tissue but are applied here to both blood and skeletal muscle, we first assessed their ability to capture age‐associated DNA methylation changes in blood (NR study) and in skeletal muscle (NR, EpiH, and Gene SMART studies). DunedinPACE, which estimates the pace of aging (years per calendar year) rather than epigenetic age, was excluded from this analysis.

In blood, all epigenetic clocks showed strong correlations with chronological age, as expected, with correlation coefficients ranging from *R* ^2^ = 0.80–0.93 (Table S1 and Figure S1). However, some clocks exhibited relatively high median absolute errors (MAE), which is an estimate of error between paired measurements, most notably PCGrimAge, with MAE values reaching up to 13.6 years.

In muscle, the muscle‐tissue specific MEAT clock performed well across all three studies (NR trial, EpiH, and Gene SMART), showing both strong correlations and low MAE values with chronological age (Table S1). GrimAge2 also effectively captured age‐related methylation changes in muscle, with high correlation (*R* ^2^ = 0.90–0.98) and moderate MAE (5.9–10.5). While PCGrimAge also correlated strongly with chronological age in muscle, its MAE ranged from 24.8 to 30.6 years, indicating that the clock overestimates age and performs poorly for absolute age estimation. The weakest performance was observed for PCHorvath, PCHannum, and PCPhenoAge, with maximum correlation coefficients around *R* ^2^ = 0.70 in the NR trial and EpiH studies, and *R* ^2^ = 0.50 in Gene SMART. Corresponding MAE values varied widely, from 9.8 years (NR PCHorvath) to 35.5 years (Gene SMART PCPhenoAge), with all clocks showing a consistent upward bias in their age estimates.

Overall, differences in MAEs across clocks reflect variability in their ability to estimate age in years, specifically in skeletal muscle, while most still capture some age‐related epigenetic changes as indicated by their moderate‐to‐high correlations with chronological age.

### 3.2 Impact of 5‐Month NR Supplementation on Epigenetic Age Acceleration in Human Blood and Skeletal Muscle

**FIGURE 2:**
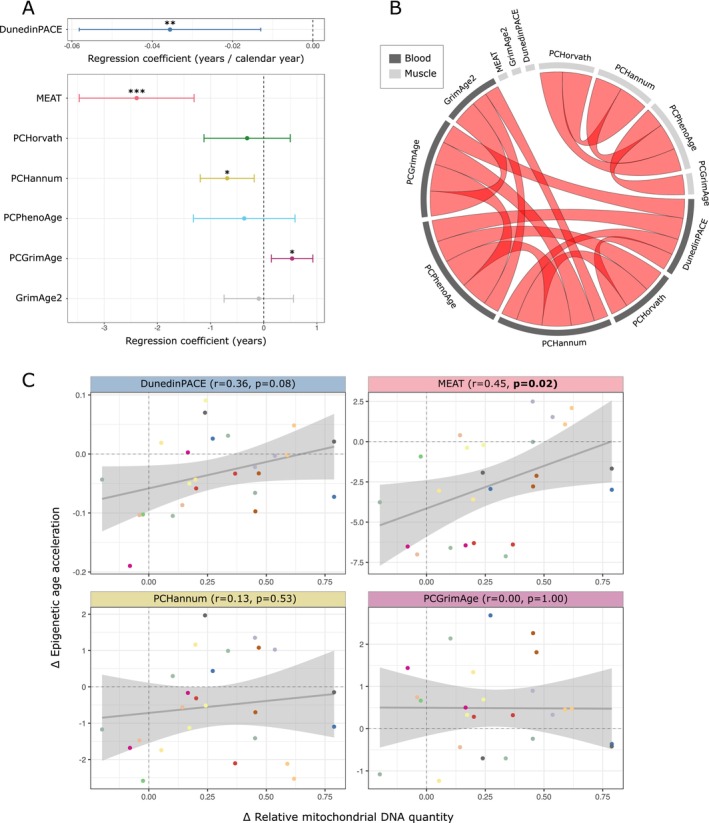
The impact of nicotinamide riboside (NR) treatment in human skeletal muscle epigenetic age acceleration (EAA). (A) Forest plots showing the effect of 5‐month NR supplementation in human skeletal muscle (*n* = 30 individuals). *p*‐values are derived from linear mixed effect models. (B) Circosplot depicting the correlations between changes in different EAA estimates after 5‐month NR supplementation derived either from blood (dark gray) or muscle tissues (light gray) within the same individual (*n* = 29 individuals). Only *p* < 0.05 correlations are shown. All observed correlations were positive. (C) Correlation between changes in EAA measures and corresponding changes in mitochondrial DNA quantity in skeletal muscle following 5‐month NR supplementation (*n* = 30 individuals). Each color represents a distinct twin pair. Δ = difference in post vs. pre values, *r* = Pearson correlation coefficient. *FDR < 0.05, **FDR < 0.01, ***FDR < 0.001.

To investigate the potential of NR supplementation to modulate epigenetic aging, we determined whether the 5‐month NR supplementation was associated with differences in the EAA measures in both blood and skeletal muscle tissues. In blood tissue (*n* = 36 individuals), we observed a significant decrease in DunedinPACE, PCHorvath, and PCHannum after 5‐months of NR supplementation (Table S2). In muscle tissue (*n* = 30 individuals), we saw that, on average, the mean EAA was lower after NR in all clocks, but PCGrimAge showed an increase in EAA (ß = 0.52, *p* = 0.007) (Figure 2A and Table 2). However, the decrease was statistically significant only when measured with DunedinPACE (ß = −0.04, *p* < 0.001), PCHannum (ß = −0.69, *p* = 0.013), and MEAT (ß = −2.39, *p* < 0.001) (Figure 2A). To investigate whether the observed changes in muscle epigenetic aging could be replicated in vitro setting with a placebo control, primary human myotubes were treated with NR or vehicle. NR exposure significantly increased intracellular NAD^+^ levels (1.52‐fold compared with control cells; Figure 2A). Mitochondrial DNA content was slightly higher in NR‐exposed cells but did not reach statistical significance (Figure 2B). No EAA measures showed a statistically significant difference between NR‐treated and control cells (Figure 2C), although the MEAT clock indicated a marginally higher epigenetic age in NR‐exposed cells (β = 1.55, *p* = 0.05). Notably, all four clocks showed effect directions opposite to those observed in the human trial.

**TABLE 2:** Epigenetic age acceleration measures at baseline and after 5‐month Nicotinamide riboside (NR) supplementation (*n* = 30 individuals), along with intra‐class correlation coefficients (ICCs) (*n* = 14 complete twin pairs) in muscle.

Clock	Mean (SD)^a^ at baseline	Mean (SD)^a^ at 5‐month	Change (SD)^a^	*p*	FDR	ICC (95% CI) baseline	ICC (95% CI) change
DunedinPACE	1.38 (0.07)	1.34 (0.06)	‐0.04 (0.06)	0.001	**0.004**	0.78 (0.4–0.92)	—
PCHorvath	0.16 (2.44)	0.03 (2.36)	−0.13 (2.00)	0.321	0.391	0.41 (0–0.75)	0.45 (0–0.78)
PCHannum	0.27 (1.60)	−0.23 (1.48)	−0.50 (1.24)	0.013	**0.023**	0.24 (0–0.66)	0.01 (0–0.54)
PCPhenoAge	0.15 (2.33)	−0.15 (2.45)	−0.30 (2.34)	0.335	0.391	0.56 (0.07–0.84)	—
PCGrimAge	−0.25 (1.67)	0.25 (1.39)	0.50 (0.95)	0.007	**0.016**	0.68 (0.23–0.89)	—
GrimAge2	0.12 (1.46)	0.05 (1.59)	−0.12 (1.79)	0.757	0.757	0.36 (0–0.73)	0.08 (0–0.58)
MEAT	0.84 (2.54)	−1.65 (3.04)	−2.49 (2.88)	3.8E‐04	**2.7E‐04**	0.66 (0.22–0.88)	0.68 (0.21–0.88)

*Note:* *p*‐Values are derived from linear mixed‐effects models adjusted for chronological age, sex, smoking, BMI, and with personID nested within familyID as a random effect. FDR < 0.05 are bolded.

Abbreviation: FDR = false discovery rate.

^a^ Units are in years, except for DunedinPACE, which is expressed in years/calendar year.

Given that our NR cohort consisted mostly of BMI‐discordant monozygotic twin pairs (*n* = 15 and 14 complete twin pairs in blood and muscle, respectively), we further investigated whether the leaner versus heavier co‐twin status influenced changes in EAA following NR in those estimates showing significant changes after NR. No significant differences in response to NR were observed between leaner and heavier co‐twins in either blood or muscle tissue (Table S3).

To quantify the relative contributions of between‐pair and within‐pair variation in EAA both at baseline and in response to NR supplementation, ICC coefficients were estimated. ICC reflects the degree of resemblance between co‐twins, with higher values indicating greater similarity. At baseline, ICCs for blood‐based EAA measures ranged from 0.86 for PCHorvath to 0.33 for PCGrimAge (Table S2). In muscle tissue, baseline ICCs ranged from 0.78 for DunedinPACE to 0.24 for PCHannum (Table 2). The ICCs for changes in EAA following NR supplementation were generally lower than those observed at baseline. Interestingly, MEAT and PCHorvath in muscle showed comparable ICCs for change and baseline values.

To assess whether changes in different EAA estimates were correlated in response to NR, we leveraged multiple epigenetic clock measures from both blood and muscle tissues in the same individuals in the NR trial. We examined correlations between changes in EAA (delta variables, i.e., post‐value minus pre‐value) across clocks and tissues. Significant correlations were observed between clocks within the same tissue, but no cross‐tissue correlations were detected (Figure 2B). Notably, the MEAT, DunedinPACE, and GrimAge2 clocks, calculated from skeletal muscle, were the only ones whose changes after NR were not correlated with any other EAA estimates.

Together, our results suggest that 5‐month NR supplementation is generally associated with reduced blood and muscle epigenetic aging similarly between leaner and heavier co‐twins, although the effects vary by both tissues and clocks.

### 3.3 Correlation Between Changes in Epigenetic Age Acceleration, and Blood NAD Metabolites and Mitochondrial DNA Quantity in Skeletal Muscle After NR Supplementation

Although, on average, both blood NAD metabolites (Lapatto et al. 2023) and skeletal muscle mtDNAq (1.36 fold higher, *p*_lmerTest_ < 0.001) and some of the EAA estimates were altered following NR supplementation, there was considerable inter‐individual variability in these changes. To explore whether changes in blood NAD metabolites and skeletal muscle mitochondrial characteristics might be associated with shifts in skeletal muscle epigenetic aging, we examined correlations between blood NAD metabolome or mtDNAq with EAA following NR supplementation. The analysis was restricted to skeletal muscle EAA, as we did not have mitochondrial measures from blood. Interestingly, changes in blood NAD metabolites including NADP and NMN, and in particular NAAD, were negatively correlated with changes in EAA derived from DunedinPACE and MEAT, respectively (Figure S3). Additionally, we noticed a positive correlation between changes in MEAT EAA and mtDNAq (*r* = 0.45, *p* = 0.02; Figure 2C) after NR, indicating that individuals with a greater increase in muscle mtDNAq tended to exhibit a larger increase in MEAT epigenetic aging. Other clocks did not show statistically significant correlations, although DunedinPACE displayed a similar trend with marginal significance (*r* = 0.36, *p* = 0.08) (Figure 2C). In the in vitro myotube experiment, we observed no correlations between EAA and intracellular NAD^+^ or mtDNAq.

We also examined within‐pair differences in changes in those pairwise correlations that were significant at the individual level to assess whether the associations persisted after accounting for shared genetic and environmental factors. Interestingly, all correlations remained statistically significant with similar or higher correlation coefficients (Table S4).

These results imply that NR induces distinct in vivo response patterns: while increased blood cell NAD^+^ biosynthesis correlates with reduced muscle epigenetic aging, elevated muscle mtDNA content instead coincides with accelerated muscle epigenetic aging. Furthermore, the associations were independent of the underlying genetic or shared environmental confounding.

### 3.4 Impact of High‐Intensity Exercise Training on Epigenetic Age Acceleration in Skeletal Muscle

**FIGURE 3:**
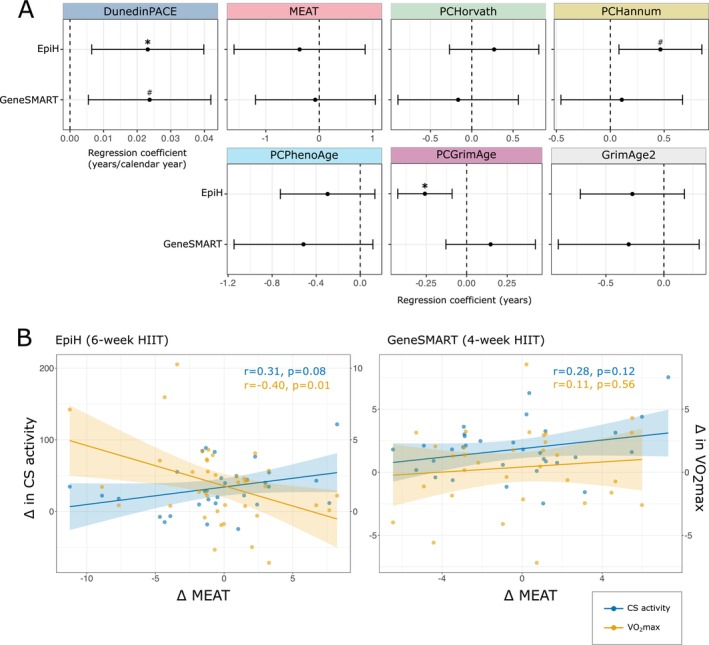
(A) Forest plot illustrating the associations between high‐intensity interval training (HIIT) and epigenetic age acceleration (EAA) across two studies. Effect sizes and 95% confidence intervals are shown. (B) Correlation between changes in MEAT‐derived EAA and changes in VO_2max_ (in yellow) and citrate synthase (CS) activity (in blue) following HIIT in the EpiH and Gene SMART studies. EpiH *n* = 39 individuals, Gene SMART *n* = 37 individuals. Δ = difference in post vs. pre values; *FDR < 0.05, #*p* < 0.05 & FDR > 0.05.

Given that exercise training has been shown to increase the skeletal muscle NAD^+^ metabolome in humans (de Guia et al. 2019), we explored whether HIIT interventions induced changes in muscle EAA using data from two studies, EpiH (*n* = 39 individuals) and Gene SMART (*n* = 37 individuals). In EpiH (6‐week HIIT intervention), out of the seven clocks that were investigated, PCGrimAge showed a significant decrease in EAA after HIIT (ß = −0.26, *p* = 0.005), and PCHannum and DunedinPACE showed an increase in EAA (ß = 0.47, *p* = 0.023 and ß = 0.02, *p* = 0.010, respectively) (Figure 3A). After a 4‐week HIIT intervention (Gene SMART), the only significant change in EAA we observed was an increase in DunedinPACE (ß = 0.02, *p* = 0.034) before versus after the exercise intervention (Figure 3A) similarly to what was observed in EpiH. Notably, the clocks that demonstrated a significant decrease in EAA upon NR showed increased EAA after HIIT (DunedinPACE). In contrast, PCGrimAge demonstrated significantly higher EAA after NR and lower EAA after HIIT (Figures 2A and 3A). The muscle‐specific MEAT clock did not change after HIIT. These results indicate that 4–6 weeks of HIIT may alter skeletal muscle epigenetic aging in directions opposite to those observed with NR supplementation in humans.

### 3.5 Correlation Between Changes in Skeletal Muscle Epigenetic Age Acceleration, Citrate Synthase Activity and Cardiorespiratory Fitness After High‐Intensity Exercise Training

Both 4‐week Gene SMART and 6‐weeks EpiH HIIT interventions have been previously shown to improve participants' VO_2max_ and increase muscle‐specific mitochondrial citrate synthase (CS) activity, an estimate of mitochondrial content in human skeletal muscle (Larsen et al. 2012; Yan et al. 2017). We investigated whether the changes in EAA were correlated with corresponding changes in VO_2max_ or CS activity in both HIIT studies. No NAD metabolome data were available for HIIT cohorts. In the EpiH study, changes in MEAT following HIIT showed a negative correlation with changes in VO_2max_, but a positive correlation with CS activity (Figure 3B). A similar positive trend between CS activity and MEAT was observed in the Gene SMART, although it did not reach statistical significance (Figure 3B). This positive association aligns with findings from the human NR trial, where MEAT changes were also positively associated with mtDNAq changes. In contrast, other epigenetic clocks demonstrated inconsistent associations across the two HIIT studies (Figure S4).

To account for the broad age range in the EpiH study, we then stratified the analyses by age into younger (age 21–42 years, *n* = 19) and older (age 55–74 years, *n* = 20) subgroups. This allowed us to determine if the younger EpiH subgroup exhibited trends more comparable to the Gene SMART cohort (age 19–47). While stratification reduced the sample size and attenuated correlation coefficients, the direction of associations remained largely consistent with the primary analysis of the entire EpiH study (Table S5). Collectively, these results suggest that HIIT‐induced changes in skeletal muscle mitochondrial content may be reflected in the MEAT clock, mirroring the pattern observed following NR supplementation.

## 4 Discussion

Given the aging population, it is essential to identify factors that promote healthy aging and biomarkers that best reflect and predict aging across tissues. Here, we use EAA as a biological aging biomarker and show that both NR supplementation and HIIT, two well‐known mitochondrial content and NAD^+^ boosting strategies, may modulate skeletal muscle epigenetic aging. Notably, these interventions appeared to work in opposite directions: NR was generally associated with reduced EAA, while HIIT was associated with higher EAA. Furthemore, changes in EAA measured by the muscle‐specific MEAT clock correlated significantly with changes in mitochondrial content across both interventions. These findings suggest that certain epigenetic clocks, including MEAT and DunedinPACE, respond to NR and HIIT interventions. Although linked to mitochondrial content and increased NAD^+^ biosynthesis, the modest correlation coefficient implies that the clocks capture unique cellular adaptations not fully explained by these markers alone. Thus, they may hold potential as tissue level biomarkers for detecting molecular responses to interventions, although the exact drivers of clock changes across tissues and individuals remain to be elucidated.

While the effects of NR on human epigenetic aging have not been widely demonstrated before, one study has shown a reduction in blood epigenetic aging following 12 weeks of NR supplementation in individuals with chronic obstructive pulmonary disease (Norheim et al. 2024). Furthermore, interventions known to modulate the NAD^+^ pathway, such as calorie restrication (CR) and enhanced diet quality, also decrease blood epigenetic aging (Waziry et al. 2023; Fitzgerald et al. 2021). Consitently, our 5‐month NR trial as significantly associated with reduced DunedinPACE in blood, alongside decreased DunedinPACE, MEAT and PCHannum in skeletal muscle. Even though we did not observe any measurable improvements in conventional metabolic parameters upon NR (Lapatto et al. 2023), the observed reduction in some of the EAA markers may indicate some early molecular adaptations that precede systemic effects. The mechanisms underlying these changes remain unclear, but may involve altered activity of NAD^+^‐dependent enzymes, including sirtuins, PARPs, and CD38, that participate in chromatin modeling, DNA methylation and mitochondrial function processes. Taken together, these findings highlight the potential of epigenetic clocks as biomarkers possibly capturing subtle cellular adaptations not reflected in standard clinical readouts, and raise the possibility that NR may promote muscle health and potentially affect aging‐related pathways.

Epigenetic age is affected by genome, environment and lifestyle factors (Reynolds et al. 2020). Using monozygotic twins, who share their genomic sequence, allowed us to explore the relative contributions of individual‐level effects versus shared genetic and environmental influences on the variability of the EAA after NR. The ICC analyses, which describe the similarity in a parameter within‐pair versus between‐pair, demonstrated that co‐twins were highly similar in most of their EAA measures at baseline, but differed more in how their EAA changed during NR supplementation. This suggests that the response to NR is primarily driven by individual‐specific factors, such as lifestyle effects or random variation, rather than shared genetic or environmental influences. Interestingly, for clocks such as MEAT and PCHorvath, which incorporate muscle‐derived methylation data in their development, we observed relatively high ICC for the change in skeletal muscle EAA. This similarity between the co‐twins (even slightly higher than at baseline) indicates that, for these clocks, the NR‐induced change in EAA is partially accounted for by genetic and shared environmental factors. This aligns with our previous finding that monozygotic twins respond similarly to NR regarding global and mitochondrial‐related DNA methylation (Lapatto et al. 2023). A previous study has suggested that NR has a more effect on EAA in those individuals with higher levels of inflammation (Norheim et al. 2024). These findings underscore an interplay between genetic and disease‐ or lifestyle‐related factors in NR supplementation responses, though defining and quantifying their individual contributions remains a significant challenge.

We have previously reported that NR supplementation can increase mitochondrial content in human muscle (Lapatto et al. 2023). However, when we mimicked the chronic effect of NR in human myotubes, 3‐day NR treatment significantly increased intracellular NAD^+^ levels, but this elevation did not translate into changes in mtDNAq or EAA measures. It may be that the direct addition of NR to myotubes has different effects than systemic administration, where interactions with other cell types are present and may be required for full mitochondrial responses absent in isolated cell cultures. Alternatively, the duration of NR exposure may have been too short to elicit detectable changes. Furthermore, the epigenetic clocks used in this study were developed using bulk tissue samples composed of multiple cell types, which may limit their applicability to homogeneous cell populations like cultured myotubes, particularly if the observed effects are primarily driven by other cell types. Other biological age estimators such as muscle‐specific protein clocks (Goeminne et al. 2025) may provide alternative insights into the effects of NR both in vivo and in vitro.

Exercise, including HIIT, is well known to confer numerous health benefits (Cartee et al. 2016), and influence skeletal muscle DNA methylation patterns (Voisin et al. 2024) and epigenetic aging markers (Ostaíza et al. 2025) to represent a more youthful methylome. In contrast, we observed that HIIT was mainly associated with increased EAA in both 4‐week Gene SMART and 6‐week EpiH studies when measured with DunedinPACE. This discrepancy with previous findings could reflect temporal molecular stress responses such as acute remodeling, inflammation, increased mitochondrial reactive oxygen species production or shifts in cellular turnover induced by exercise required for long‐term adaptations. Additionally, changes in epigenetic age in skeletal muscle induced by exercise may be driven by shifts in cell‐type composition (Lovrić et al. 2022), rather than a change in the aging rate of a single cell type. Furthermore, the effects of HIIT were generally opposite to those observed after NR supplementation. Clocks that showed decreased EAA after NR supplementation—such as DunedinPACE—showed increased EAA following HIIT, while others like PCGrimAge showed the reverse pattern. This divergence suggests that NR and HIIT may influence distinct epigenetic pathways, reflect differences in the duration of these interventions or capture short‐term cellular strain or inflammatory responses potentially differing between NR and HIIT. As a result, interpretation of these scores remains limited, and increases in EAA should not be automatically interpreted as increased aging per se. Longitudinal studies are therefore essential to clarify how epigenetic scores evolve in response to different biological stimuli.

Increased mitochondrial content and activity and NAD^+^ levels have been generally associated with positive outcomes, including with lower epigenetic age (Kabacik et al. 2022). In line, the increase in blood NAD metabolites correlated with the reduced muscle EAA in our study, suggesting that NR may promote favorable metabolic adaptations associated with reduced EAA. In contrast, we observed a positive correlation between changes in skeletal muscle mtDNAq and MEAT EAA, indicating that individuals with greater increases in mtDNAq after NR tended to exhibit smaller reductions in EAA. Similarly, following HIIT, MEAT‐derived EAA was positively correlated with changes in CS activity, another marker of mitochondrial content. One potential explanation is that individuals with greater increases in mtDNA and CS activity may be in a more active phase of muscle regeneration and remodeling, which can involve transient cellular stress (Lee et al. 2000) that may at least temporarily elevate MEAT EAA. Alternatively, these relationships may reflect the influence of other biological processes, such as inflammation, which can affect both EAA and mitochondrial markers and CS activity (Franceschi et al. 2018). Furthermore, VO_2max_ correlated with MEAT‐derived EAA only in the EpiH intervention, where we observed larger improvements in VO_2max_ than in the GeneSMART, suggesting that improvements in cardiorespiratory fitness may be linked to EAA. However, these interpretations remain speculative, and the underlying mechanisms require further investigation. Overall, these findings suggest that epigenetic aging in muscle is linked to cellular stress and metabolic changes, but cannot be explained by mitochondrial changes alone, highlighting the complex biology behind epigenetic aging.

Blood‐based epigenetic clocks may not fully capture aging‐related changes in other tissues. While we previously showed that blood‐based clocks are weaker in detecting muscle aging and physical function (Sillanpää et al. 2021), we aimed to examine how these clocks respond to different interventions within the same individual and evaluate their applicability. Blood‐developed clocks often showed high deviations from chronological age when calculated from muscle but were moderately to highly correlated with age, suggesting that although some CpGs may not reflect literal muscle age, these clocks still capture relevant age‐related signatures shared across tissues. In contrast, the MEAT clock demonstrated superior accuracy and responsiveness, reinforcing the importance of tissue‐targeted clocks in aging research. Furthermore, we observed no cross‐tissue correlations between clocks within individuals after NR, even for those showing significant decreases in both tissues such as DunedinPACE, which further underscores the tissue‐ and clock‐specific nature of epigenetic aging. Future work should examine the epigenetic signatures driving these clock‐specific changes after NR and HIIT and their links to underlying biological processes.

This study has several limitations. First, modest sample sizes may limit statistical power and generalizability, particularly given the known random biological and technical variation in EAA estimates, contributing to limited individual replicability. Second, the NR and HIIT interventions lacked placebo controls, making it difficult to rule out confounding effects or natural fluctuations in EAA over time. Third, heterogeneity existed across studies in intervention lengths, participant characteristics, and mitochondrial measurement methodologies and methylation array versions (EPIC v1 vs. v2) likely contributing to discrepancies. Lastly, although we aimed to mimic chronic NR treatment in human myotubes, experimental conditions differed substantially from in vivo culture. Despite these challenges, data from controlled human interventions with access to the primary target tissue, rather than blood‐based surrogates only, offer unique and robust perspective into tissue‐specific epigenetic aging in response to NR supplementation and HIIT.

Our findings provide novel insights by demonstrating that both NR supplementation and exercise can modulate epigenetic aging in muscle, and that these effects appear to be both clock‐ and intervention‐specific. While this study is limited with the mechanistic data, it provides evidence for future research aimed at disentangling the molecular mechanisms underlying fluctuations in human biological aging in response to mitochondria targeting interventions. Ideally, such work should incorporate studies with placebo‐controlled and longitudinal designs with larger sample sizes. These designs, combined with clinically meaningful endpoints, will be essential to determine if and how changes in epigenetic age relate to mitochondrial function, NAD^+^ metabolism, and ultimately, age‐related functional decline and disease risk.

## Author Contributions

A.H. and M.O. conceptualized and designed the study. A.H. preprocessed the data, performed statistical analyses, visualized the results, and wrote the first draft of the manuscript. R.K. and E.P. contributed to the conceptualization of the myotube cell culture experiment. L.U.‐K. conducted the myotube cell culture experiment. S.A. contributed to the interpretation of findings and offered theoretical guidance on exercise research. J.K., B.W.K., S.H., K.H.P., and E.P. conceived, collected, and generated data for the NR trial study. I.B., J.W.H., L.G., R.S., and S.L. conceived, collected, and generated data for the EpiH study. M.J., R.G., and N.E. conceived, collected, and generated data for the Gene SMART study. All authors provided critical feedback on the manuscript and approved the final version for submission.

## Funding

The study was supported by the Research Council of Finland (335445, 314455 for EP, and 328685, 307339, 297908, 251316 for MO, 272376, 266286, 314383, 335443, 369181 for KHP, 361956, 338417 for SH, 336823, 352792 for JK), Research Council of Finland Profi6 funding (336449) awarded to the University of Oulu (E.P.), Finnish Diabetes Research Foundation (E.P., S.H., K.P.), the Minerva Foundation (M.O.), the Liv och Hälsa society (M.O.), the Sigrid Juselius Foundation (M.O., K.H.P.), The Finnish Cultural Foundation Kymenlaakso Fund (M.O. and A.H.), Orion Foundation (S.H., BWvdK), Finnish Medical Foundation (K.H.P., S.H.), Finnish Foundation for Cardiovascular Research (K.H.P.), Novo Nordisk Foundation (NNF10OC1013354, NNF17OC0027232, NNF20OC0060547, NNF24OC0091683, NNF25SA0103783 for K.H.P.; NNF23SA0083953 and NNF25OC0100827 for S.H., NNF24SA0090438 for B.K.), Paulo Foundation (S.H.), Gyllenberg Foundation (K.H.P.), Paavo Nurmi Foundation (S.H.), Helsinki University Hospital (S.H., K.H.P.), Government Research Funds (K.H.P., S.H.), University of Helsinki (K.H.P), Juho Vainio Foundation (S.A.), and Yrjö Jahnsson Foundation (B.K.).

## Ethics Statement

The NR trial study protocol was approved by the Ethics Committee of the Helsinki University Central Hospital (protocol number 270/13/01/2008). The EpiH study was approved by the Ethical Committee of Copenhagen (H‐3‐2012‐024). The Gene SMART study was approved by the Victoria University Human Ethics Committee (HRE13‐223).

## Consent

Written informed consent was obtained from all participants of each study.

## Conflicts of Interest

The authors declare no conflicts of interest.

## Supporting information


**Figure S1:** Correlation between chronological age (X‐axis) and estimated epigenetic age (Y‐axis) in (A) blood tissue of the NR cohort and skeletal muscle of (B) the NR trial, (C) EpiH, and (D) Gene SMART cohorts. The red/green lines indicate the fitted linear lines (red for blood, green for muscle) whereas the dashed line indicates the identity (x = y) line. Mean absolute error (MAE) and Pearson correlation coefficients are presented in each plot.
**Figure S2:** (A) Levels of NAD+, (B) mitochondrial DNA quantity (mtDNAq) and (C) epigenetic age acceleration measures in control vs. NR‐treated cells at the three‐day timepoint (*n* = 6 replicates per group). *p*‐values are derived from Wilcoxon signed‐rank tests (A,B) or from linear models (C).
**Figure S3:** Correlations between changes skeletal muscle epigenetic age (Y‐axis) and changes in blood NAD+ metabolites (Y‐axis) after 5‐month of NR. *n* = 23–25 individuals, *p‐value < 0.05, **p‐value < 0.01. ADPR = adenosine diphosphate ribose, Me4Py = *N*‐methyl‐4‐pyridone‐5‐carboxamide, NAAD = nicotinic acid adenine dinucleotide, NADP = nicotinamide adenine dinucleotide phosphate, NAR = nicotinic acid riboside, NMN = nicotinamide mononucleotide.
**Figure S4:** Correlations between changes in EAA and changes in citrate synthase (CS) activity and cardiorespiratory fitness (VO₂_max_) following HIIT in the EpiH and Gene SMART cohorts. Each point represents a correlation coefficient for one cohort, with gray lines connecting paired estimates to illustrate differences between cohorts. Point shapes indicate significance levels based on *p*‐values. Δ = post value minus pre value, ns = not significant.
**Table S1:** Person correlation coefficients (*R* ^2^) and mean absolute error (MAE) between epigenetic ages and chronological ages across all human cohorts in the study.
**Table S2:** Epigenetic age acceleration measures at baseline and after 5‐month NR supplementation (*n* = 36 individuals), along with intra‐class correlation coefficients (ICCs) (*n* = 15 complete twin pairs) in blood. *p*‐values are derived from linear mixed‐effects models adjusted for chronological age, sex, smoking and BMI, with personID nested within familyID as a random effect. FDR < 0.05 are bolded.
**Table S3:** Differences in NR‐induced changes in epigenetic age acceleration (EAA) in BMI‐discordant monozygotic twin pairs, comparing leaner co‐twins to their heavier counterparts (reference group) in muscle (*n* = 14 pairs) and blood (*n* = 15 pairs). Only clocks that showed a significant main effect of NR are shown.
**Table S4:** Correlation between within‐pair changes in skeletal muscle epigenetic age acceleration and changes in blood NAD metabolites or skeletal muscle mitochondrial DNA quantity (mtDNAq) after NR supplementation. Only comparisons that showed significant correlation in the individual‐level analyses were presented. *p*‐values < 0.05 are bolded.
**Table S5:** Pearson correlation between changes in epigenetic age acceleration (EAA), and citrate synthase (CS) activity and VO_2max_ after a 6‐week high‐intensity interval training (HIIT) intervention, stratified by younger (*n* = 19; age 21–42 years) and older (*n* = 20; age 55–74 years) participants in the EpiH cohort. *p*‐values < 0.05 are bolded.

## Data Availability

The FTC DNA methylation is part of the ‘Twin Study’ and deposited with the Biobank of the Finnish Institute for Health and Welfare (https://thl.fi/en/research‐and‐development/thl‐biobank/for‐researchers/sample‐collections/twin‐study). For details on accessing the data, see: https://thl.fi/en/research‐and‐development/thl‐biobank/for‐researchers/application‐process. The EpiH DNA methylation data is in the process of being deposited into a public repository. In the meantime, the data can be requested from the corresponding author. The Gene SMART DNA methylation data is deposited to Gene Expression Omnibus with accession code GSE171140.
