# Supplementary material for: The Divergent Effects of Nicotinamide Riboside and High‐Intensity Exercise Training on Skeletal Muscle Epigenetic Aging
Source: Aging Cell. 2026 Jul 21;25(8):e70638. doi: 10.1111/acel.70638 (PMC13388492; doi:10.1111/acel.70638)
Supplement: Supplementary file 1 — Figure S1: Correlation between chronological age (X‐axis) and estimated epigenetic age (Y‐axis) in (A) blood tissue of the NR cohort and skeletal muscle of (B) the NR trial, (C) EpiH, and (D) Gene SMART cohorts. The red/green lines indicate the fitted linear lines (red for blood, green for muscle) whereas the dashed line indicates the identity (x = y) line. Mean absolute error (MAE) and Pearson correlation coefficients are presented in each plot. Figure S2: (A) Levels of NAD+, (B) mitochondrial DNA quantity (mtDNAq) and (C) epigenetic age acceleration measures in control vs. NR‐treated cells at the three‐day timepoint (n = 6 replicates per group). p‐values are derived from Wilcoxon signed‐rank tests (A,B) or from linear models (C). Figure S3: Correlations between changes skeletal muscle epigenetic age (Y‐axis) and changes in blood NAD+ metabolites (Y‐axis) after 5‐month of NR. n = 23–25 individuals, *p‐value < 0.05, **p‐value < 0.01. ADPR = adenosine diphosphate ribose, Me4Py = N‐methyl‐4‐pyridone‐5‐carboxamide, NAAD = nicotinic acid adenine dinucleotide, NADP = nicotinamide adenine dinucleotide phosphate, NAR = nicotinic acid riboside, NMN = nicotinamide mononucleotide. Figure S4: Correlations between changes in EAA and changes in citrate synthase (CS) activity and cardiorespiratory fitness (VO₂max) following HIIT in the EpiH and Gene SMART cohorts. Each point represents a correlation coefficient for one cohort, with gray lines connecting paired estimates to illustrate differences between cohorts. Point shapes indicate significance levels based on p‐values. Δ = post value minus pre value, ns = not significant. Table S1: Person correlation coefficients (R 2) and mean absolute error (MAE) between epigenetic ages and chronological ages across all human cohorts in the study. Table S2: Epigenetic age acceleration measures at baseline and after 5‐month NR supplementation (n = 36 individuals), along with intra‐class correlation coefficients (ICCs) (n = 15 complete twin pa [file ACEL-25-e70638-s001.docx]

**Supporting Information**

**Supplementary Tables**

**Supplementary Table 1.** Person correlation coefficients (R2) and mean absolute error (MAE) between epigenetic ages and chronological ages across all human cohorts in the study.

|  | **NR trial** | | | | **EpiH** | | **Gene SMART** | |
| --- | --- | --- | --- | --- | --- | --- | --- | --- |
|  | **Blood** | | **Muscle** | | **Muscle** | | **Muscle** | |
|  | **R2** | **MAE** | **R2** | **MAE** | **R2** | **MAE** | **R2** | **MAE** |
| PCHorvath | 0.902 | 7.201 | 0.719 | 9.894 | 0.709 | 13.006 | 0.540 | 15.189 |
| PCHannum | 0.929 | 10.690 | 0.717 | 14.577 | 0.721 | 13.570 | 0.562 | 20.925 |
| PCPhenoAge | 0.801 | 4.599 | 0.690 | 30.356 | 0.784 | 20.506 | 0.530 | 35.232 |
| PCGrimAge | 0.920 | 13.636 | 0.976 | 27.474 | 0.993 | 24.847 | 0.966 | 30.557 |
| GrimAgev2 | 0.813 | 8.455 | 0.956 | 6.545 | 0.980 | 5.894 | 0.903 | 10.492 |
| MEAT* | - | - | 0.937 | 2.618 | 0.969 | 3.255 | 0.880 | 3.582 |

* MEATv2 was used in all other cohorts except for Gene SMART

**Supplementary Table 2. Epigenetic age acceleration measures at baseline and after 5-month NR supplementation (n=36 individuals), along with intra-class correlation coefficients (ICCs) (n=15 complete twin pairs) in blood.** P-values are derived from linear mixed-effects models adjusted for chronological age, sex, smoking and BMI, with personID nested within familyID as a random effect. FDR<0.05 are bolded.

| **Clock** | **Mean (SD)* at baseline** | **Mean (SD)* at 5-month** | **Change (SD)*** | **P-value** | **FDR** | **ICC (95% CI) baseline** | **ICC (95% CI) change** |
| --- | --- | --- | --- | --- | --- | --- | --- |
| DunedinPACE | 0.96 (0.10) | 0.94 (0.09) | -0.02 (0.05) | 0.050 | 0.101 | 0.55  (0.11-0.83) | - |
| PCHorvath | 0.51 (2.86) | -0.58 (2.92) | -1.09 (1.67) | 3.0E-04 | **0.002** | 0.86  (0.63-0.95) | 0.26  (0-0.66) |
| PCHannum | 0.31 (2.46) | -0.37 (2.76) | -0.68 (1.38) | 0.011 | **0.034** | 0.64  (0.21-0.86) | 0.42  (0-0.76) |
| PCPhenoAge | -0.15 (5.04) | -0.20 (5.20) | -0.05 (2.79) | 0.865 | 0.865 | 0.60  (0.14-0.85) | 0.69  (0.27-0.89) |
| PCGrimAge | -0.24 (2.59) | 0.00 (2.86) | 0.24 (1.24) | 0.291 | 0.437 | 0.33  (0-0.68) | 0.28  (0-0.68) |
| GrimAge2 | -0.27 (4.10) | 0.02 (4.31) | 0.29 (1.92) | 0.401 | 0.482 | 0.48  (0-0.78) | 0.13  (0-0.6) |

* Units are in years, except for DunedinPACE, which is expressed in years/year.

**Supplementary Table 3. Differences in NR-induced changes in epigenetic age acceleration (EAA) in BMI-discordant monozygotic twin pairs, comparing leaner co-twins to their heavier counterparts (reference group) in muscle (n = 14 pairs) and blood (n= 15 pairs).** Only clocks that showed a significant main effect of NR are shown.

| **Tissue** | **Clock** | **Coefficient** | **P-value** |
| --- | --- | --- | --- |
| Muscle | DunedinPACE | 0.012 | 0.664 |
|  | PCHannum | 0.610 | 0.167 |
|  | PCGrimAge | 0.176 | 0.675 |
|  | MEAT | -0.060 | 0.936 |
| WBC | DunedinPACE | -0.014 | 0.413 |
|  | PCHannum | -0.361 | 0.381 |
|  | PCHorvath | -0.416 | 0.379 |

**Supplementary Table 4. Correlation between within-pair changes in skeletal muscle epigenetic age acceleration and changes in blood NAD metabolites or skeletal muscle mitochondrial DNA quantity (mtDNAq) after NR supplementation.** Only comparisons that showed significant correlation in the individual-level analyses were presented. P-values<0.05 are bolded.

| **Clock** | **NAD metabolite / mtDNAq** | **Pearson correlation** | **P-value** | **Number of pairs** |
| --- | --- | --- | --- | --- |
| DunedinPACE | NADP | -0.484 | **0.042** | 9 |
| DunedinPACE | NMN | -0.619 | **0.008** | 8 |
| MEAT | NAAD | -0.646 | **0.004** | 9 |
| MEAT | mtDNAq | 0.496 | **0.014** | 12 |

**Supplementary Table 5. Pearson correlation between changes in epigenetic age acceleration (EAA), and citrate synthase (CS) activity and VO_2max_ after a 6-week high-intensity interval training (HIIT) intervention, stratified by younger (n = 19; age 21–42 years) and older (n = 20; age 55–74 years) participants in the EpiH cohort.** P-values<0.05 are bolded.

|  | **EAA** | **Cor (old)** | **Cor (young)** | **P.value (old)** | **P.value (old)** |
| --- | --- | --- | --- | --- | --- |
| CS activity | DunedinPACE | 0.087 | -0.157 | 0.750 | 0.575 |
|  | GrimAge2 | 0.033 | -0.110 | 0.902 | 0.697 |
|  | MEAT | 0.223 | 0.294 | 0.407 | 0.287 |
|  | PCGrimAge | 0.128 | -0.579 | 0.636 | **0.024** |
|  | PCHannum | -0.179 | -0.454 | 0.507 | 0.089 |
|  | PCHorvath | -0.252 | -0.466 | 0.347 | 0.080 |
|  | PCPhenoAge | -0.275 | -0.347 | 0.302 | 0.205 |
| VO_2max_ | DunedinPACE | 0.194 | 0.246 | 0.426 | 0.296 |
|  | GrimAge2 | -0.457 | -0.034 | 0.049 | 0.885 |
|  | MEAT | -0.413 | -0.414 | 0.078 | 0.070 |
|  | PCGrimAge | 0.155 | -0.047 | 0.526 | 0.844 |
|  | PCHannum | 0.268 | 0.017 | 0.267 | 0.942 |
|  | PCHorvath | 0.112 | 0.338 | 0.647 | 0.145 |
|  | PCPhenoAge | 0.059 | 0.129 | 0.811 | 0.587 |

**Supplementary Figures**

**Supplementary Figure 1.** Correlation between chronological age (X-axis) and estimated epigenetic age (Y-axis) in A) blood tissue of the NR cohort and skeletal muscle of B) the NR trial, C) EpiH, and D) Gene SMART cohorts. The red/green lines indicate the fitted linear lines (red for blood, green for muscle) whereas the dashed line indicates the identity (x=y) line. Mean absolute error (MAE) and Pearson correlation coefficients are presented in each plot.

**Supplementary Figure 2.** A) Levels of NAD+, B) mitochondrial DNA quantity (mtDNAq) and C) epigenetic age acceleration measures in control vs. NR-treated cells at the three-day timepoint (n= 6 replicates per group). P-values are derived from Wilcoxon signed-rank tests (A,B) or from linear models (C).

**Supplementary Figure 3.** Correlations between changes skeletal muscle epigenetic age (Y-axis) and changes in blood NAD+ metabolites (Y-axis) after 5-month of NR.
n = 23-25 individuals, * p-value <0.05, **p-value<0.01
NAAD = nicotinic acid adenine dinucleotide; NADP = nicotinamide adenine dinucleotide phosphate; NMN = nicotinamide mononucleotide; ADPR = adenosine diphosphate ribose; NAR = nicotinic acid riboside; Me4Py = N-methyl-4-pyridone-5-carboxamide

**Supplementary Figure 4.** Correlations between changes in EAA and changes in citrate synthase (CS) activity and cardiorespiratory fitness (VO₂_max_) following HIIT in the EpiH and Gene SMART cohorts. Each point represents a correlation coefficient for one cohort, with grey lines connecting paired estimates to illustrate differences between cohorts. Point shapes indicate significance levels based on p-values.

Δ = post value minus pre value, ns = not significant

**Supplementary Methods**

Myotube cell culture

Myoblasts were cultured in high-glucose DMEM (Gibco, 10313-021) supplemented with 20% FBS (Gibco, A5256701), 2 mM L-glutamine (Gibco, 51500-056), 100 U/mL penicillin, and 100 µg/mL streptomycin (Gibco, 15140-122) and differentiated to myotubes by changing the media to high-glucose DMEM supplemented with 2% horse serum (Gibco, 26050-088), 1% Insulin-Transferrin-Selenium-Ethanolamine (ITS-X) supplement (Gibco, 51500-056), 2 mM L-glutamine, 100 U/mL penicillin and 100 ug/mL streptomycin.
